# The Annotation of RNA Motifs

**DOI:** 10.1002/cfg.213

**Published:** 2002-12

**Authors:** Neocles B. Leontis, Eric Westhof

**Affiliations:** 1 Chemistry Department and Center for Biomolecular Sciences Overman Hall Bowling Green State University Bowling Green OH 43403 USA; 2 Institut de Biologie Moléculaire et Cellulaire du CNRS Modélisation et Simulations des Acides Nucléiques UPR 9002 15 rue René Descartes Strasbourg Cedex F-67084 France

## Abstract

The recent deluge of new RNA structures, including complete atomic-resolution views of both subunits of the ribosome, has on the one hand literally overwhelmed our individual abilities to comprehend the diversity of RNA structure, and on the other hand presented us with new opportunities for comprehensive use of RNA sequences for comparative genetic, evolutionary and phylogenetic studies. Two concepts are key to understanding RNA structure: hierarchical organization of global structure and isostericity of local interactions. Global structure changes extremely slowly, as it relies on conserved long-range tertiary interactions. Tertiary RNA–RNA and quaternary RNA–protein interactions are mediated by RNA motifs, defined as recurrent and ordered arrays of non-Watson–Crick base-pairs. A single RNA motif comprises a family of sequences, all of which can fold into the same three-dimensional structure and can mediate the same interaction(s). The chemistry and geometry of base pairing constrain the evolution of motifs in such a way that random mutations that occur within motifs are accepted or rejected insofar as they can mediate a similar ordered array of interactions. The steps involved in the analysis and annotation of RNA motifs in 3D structures are: (a) decomposition of each motif into non-Watson–Crick base-pairs; (b) geometric classification of each basepair; (c) identification of isosteric substitutions for each basepair by comparison to isostericity matrices; (d) alignment of homologous sequences using the isostericity matrices to identify corresponding positions in the crystal structure; (e) acceptance or rejection of the null hypothesis that the motif is conserved.


**Comparative and Functional Genomics**
*Comp Funct Genom* 2002; **3**: 518–524.
Published online in Wiley InterScience (www.interscience.wiley.com). **DOI:** 10.1002/cfg.213

**Conference Review**

### The annotation of RNA motifs

Neocles B. Leontis[1]* and Eric Westhof[2]**

[1] *Chemistry Department and Center for Biomolecular Sciences, Overman Hall, Bowling Green State University, Bowling Green, OH 43403, USA*
[2] *Institut de Biologie Moléculaire et Cellulaire du CNRS, Modélisation et Simulations des Acides Nucléiques, UPR 9002, 15 rue René Descartes, F-67084 Strasbourg Cedex, France*

*Correspondence to either:*

**Neocles B. Leontis, Chemistry Department and Center for Biomolecular Sciences, Overman Hall, Bowling Green State University, Bowling Green, OH 43403, USA.*
*E-mail: Leontis@bgnet.bgsu.edu*

*or*

***Eric Westhof, Institute de Biologie Moléculaire et Cellulaire du CNRS, Modélisation et Simulations des Acides Nucléiques, UPR 9002, 15 rue René Descartes, F-67084 Strasbourg Cedex, France.*
*E-mail: e.westhof@ibmc.u-strasfg.fr*

Received: 9 September 2002
Accepted: 1 October 2002

### Abstract

**The recent deluge of new RNA structures, including complete atomic-resolution views of both subunits of the ribosome, has on the one hand literally overwhelmed our individual abilities to comprehend the diversity of RNA structure, and on the other hand presented us with new opportunities for comprehensive use of RNA sequences for comparative genetic, evolutionary and phylogenetic studies. Two concepts are key to understanding RNA structure: hierarchical organization of global structure and isostericity of local interactions. Global structure changes extremely slowly, as it relies on conserved long-range tertiary interactions. Tertiary RNA–RNA and quaternary RNA–protein interactions are mediated by RNA motifs, defined as recurrent and ordered arrays of non-Watson–Crick base-pairs. A single RNA motif comprises a family of sequences, all of which can fold into the same three-dimensional structure and can mediate the same interaction(s). The chemistry and geometry of base pairing constrain the evolution of motifs in such a way that random mutations that occur within motifs are accepted or rejected insofar as they can mediate a similar ordered array of interactions. The steps involved in the analysis and annotation of RNA motifs in 3D structures are: (a) decomposition of each motif into non-Watson–Crick base-pairs; (b) geometric classification of each basepair; (c) identification of isosteric substitutions for each basepair by comparison to isostericity matrices; (d) alignment of homologous sequences using the isostericity matrices to identify corresponding positions in the crystal structure; (e) acceptance or rejection of the null hypothesis that the motif is conserved. Copyright © 2002 John Wiley & Sons, Ltd.**

**Keywords:** **RNA motif; annotation; non-Watson–Crick basepair; shallow-groove; sugar-edge; Watson–Crick edge; Hoogsteen edge; isostericity matrix**

### Introduction

Nucleic acid bases interact by stacking or by hydrogen bonding edge-to-edge. Stacking interactions provide most of the driving force for folding, while hydrogen bonding provides directionality and specificity to the interactions. The regular A-form RNA double helix is due to the remarkable isostericity of the standard or canonical Watson–Crick pairs that allows each of the four combinations to substitute for any of the others without distorting the three-dimensional helical structure. The canonical Watson–Crick pairs, however, represent only one of many possible edge-to-edge interactions [9, 11, 12]. The rapid progress of RNA crystallography has revealed a rich variety of base-pairing geometries and thus of complex tertiary structural motifs [3, 15].

While only about 60–70% of bases in structured RNAs are base-paired in canonical Watson–Crick fashion, most of the rest participate in some other kind of edge-to-edge interactions with one or more other bases. This is borne out

Copyright © 2002 John Wiley & Sons, Ltd.

in the atomic-resolution structures of the large and small ribosomal subunits, the solution of which has expanded our database of RNA structure several-fold [1, 2, 4, 8]. The non-Watson–Crick pairs define, in large part, the tertiary structure of an RNA. Thus, the tertiary structure can be decomposed into a collection of 3D contacts, some of them being promoted by 3D motifs that are held together by pairwise interactions. The base–base contacts can then be specified simply by indicating the interacting edges and the relative orientations of the glycosidic bonds of the two bases. Motif identification and analysis begins with classification of all base-pairs in a structure. Base triples are decomposed into two (sometimes three) pairs.

First, it will be recalled that there are 12 basic families of base-pairs and examples from each family will be illustrated schematically [9, 12]. Then, our conventions for annotating motifs will be demonstrated and the utility of the nomenclature in summarizing RNA tertiary structure in a 2D format will be illustrated. Examples of observed and modelled base-pairs, accompanied by isostericity matrices for each geometric family, can be viewed on the websites: **http://www.bgsu.edu/departments/chem/RNA/pages/** and **http://www-ibmc.u-strasbg.fr/upr-9002/westhof/**

### Twelve basic geometric families

RNA purine and pyrimidine bases present three edges for H-bonding interactions, as shown for a representative base in Figure 1. The three edges are labelled the *Watson–Crick edge*, the *Hoogsteen edge* and the *Sugar edge* (the latter includes the 2′-hydroxyl group when the nucleotide is in the *anti* configuration.) In the right panel of Figure 1, a triangle is used to represent a nucleotide and the corresponding edges are labelled. In each triangle, the sides adjacent to the right angle represent the Watson–Crick and Sugar edges of each base, while the hypotenuse of the triangle represents the Hoogsteen edge. A cross or circle in the corner where the Hoogsteen and Sugar edges meet indicates the orientation of the sugar-phosphate backbone relative to the plane of the page (5′ to 3′ or 3′ to 5′). Although ‘Hoogsteen edge’ applies only to purines, it is also used to refer also to the CH edge of pyrimidines, as the atoms involved are normally found in the *deep (major) groove* of the A-type helix.

A given edge of one base can potentially interact in a plane with any one of the three edges of a second base, and can do so in either the *cis* or *trans* orientation of the glycosidic bonds. The *cis* and *trans* orientations follow the usual stereochemical meanings. The 12 possible, distinct edge-to-edge base-pairing geometries are illustrated in Figure 2, using the triangle representation for the bases. The upper row illustrates the six distinct *cis* pairings and the lower row the six *trans* pairings, each one positioned below the corresponding *cis* pair. Each pairing geometry is designated by stating the interacting edges of the two bases (*Watson–Crick*, *Hoogsteen* or *Sugar* edge) and the relative glycosidic bond orientation, *cis* or *trans*. A historically based priority rule is invoked for listing the bases in a pair: Watson–Crick edge > Hoogsteen edge > Sugar edge. The 12 base pair geometries are listed in Table 1, with the local strand orientations in the default *anti* configurations of the bases with respect to the sugars.

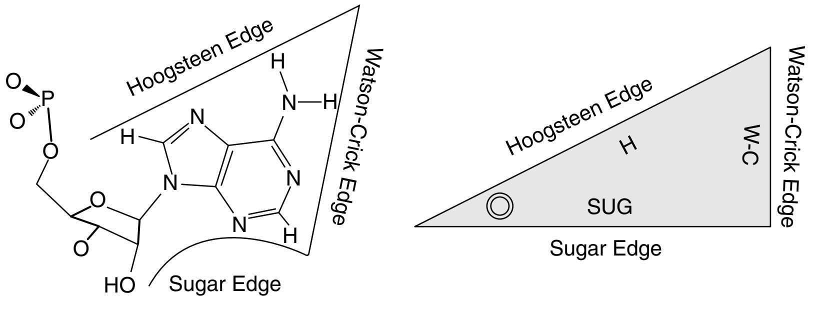

**Figure 1.** (Left) Chemical structure of a purine nucleotide illustrating the three edges available for base-to-base interaction. (Right) Representation of an RNA base as a triangle, with edges labelled as in Figure 2. For more details see [9, 12]

Copyright © 2002 John Wiley & Sons, Ltd. 

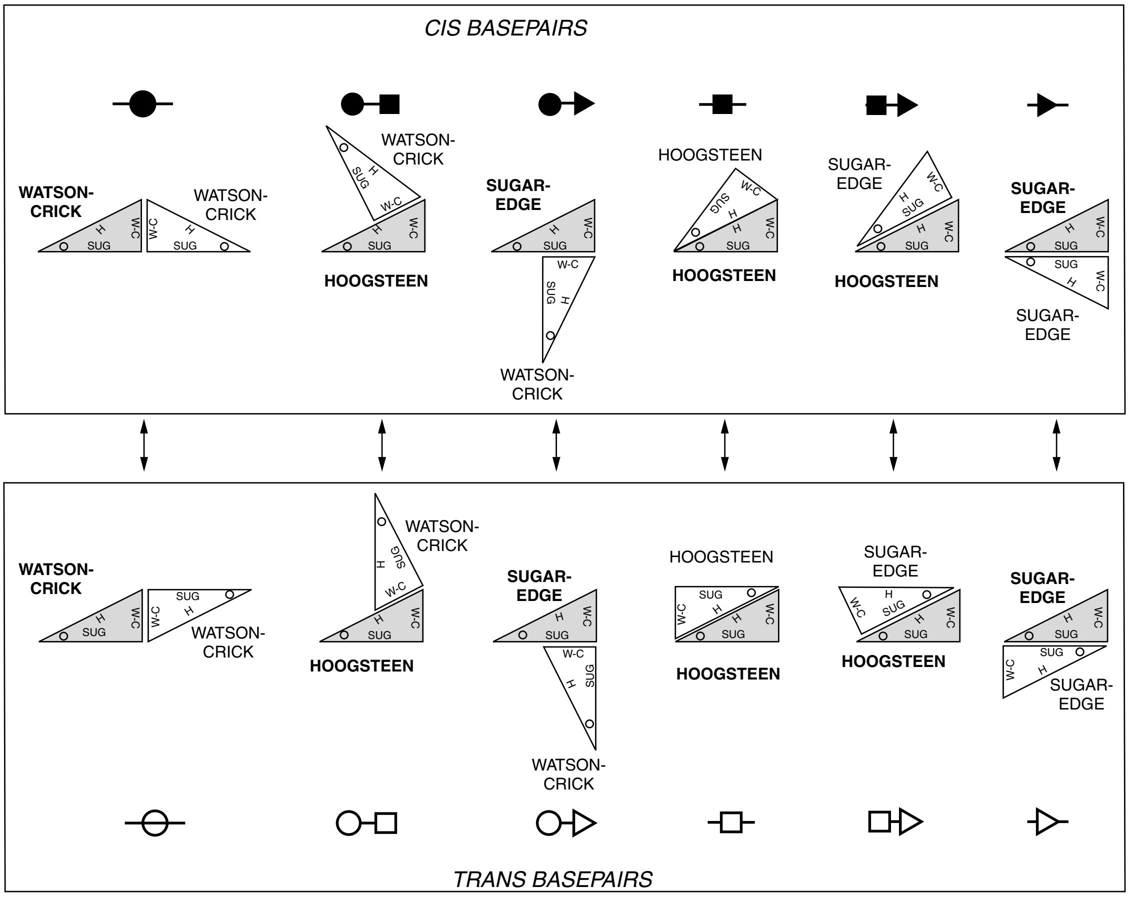

**Figure 2.** (Upper) Six possible *cis* base-pairing geometries. (Lower) Six possible *trans* base-pairing geometries

### Annotation of 2D diagrams

Accurate and unambiguous annotation of RNA motifs on standard 2D drawings allows one to communicate succinctly the essential features of a motif. This, in turn, facilitates recognition of shared 3D tertiary motifs and foldings. What are the essential elements of such drawings, which can furthermore be coded easily and used for computer aided motif identification? Such diagrams should indicate:

1. The classical secondary structure (contiguous canonical pairs forming A-form double-stranded helices maintained by Watson–Crick and wobble pairs).
2. All non-Watson–Crick pairs and the geometric family to which they belong, designated using unique symbols.
3. All points in the covalent chain at which the strand polarity reverses direction.
4. Key base stacking interactions, to the degree possible without overly cluttering the picture.
5. Sequential numbering of nucleotides (5′ to 3′) to aid in tracing the covalent chain.
6. Which nucleotides adopt the less usual *syn* conformation about the glycosidic bond.

Nucleotides can be indicated by single black, capital letters (A, G, C or U) as usual. Bold or red-coloured fonts are suggested to indicate which bases are in the less usual *syn* configuration of the glycosidic bond. To designate canonical Watson–Crick and wobble pairs, one can use the symbols — for *both* AU and GC pairs and • for the wobble GU pair [5], but the convention — for AU pairs, = for GC pairs, and ∘ for GU wobble pairs is more explicit [13] and allows

Copyright © 2002 John Wiley & Sons, Ltd.

**Table 1.** The 12 geometric families of nucleic acid base pairs with symbols for annotating secondary structure diagrams [12]. The local strand orientation is given in the last column, assuming that all bases are in the default *anti* conformation; a *syn* orientation would imply a reversal of orientation; for the global orientation, the stereochemistry at the phosphate groups has to be considered. In the very rare case that both bases are *syn*, the strand orientations revert to those given in the table [14]

| No. | Glycosidic bond orientation | Interacting edges | Symbol | Default local strand orientation |
|---|---|---|---|---|
| 1 | *cis* | Watson–Crick/Watson–Crick | –●– | Anti-parallel |
| 2 | *trans* | Watson–Crick/Watson–Crick | –○– | Parallel |
| 3 | *cis* | Watson–Crick/Hoogsteen | ●–■ | Parallel |
| 4 | *trans* | Watson–Crick/Hoogsteen | ○–□ | Anti-parallel |
| 5 | *cis* | Watson–Crick/Sugar edge | ●–▶ | Anti-parallel |
| 6 | *trans* | Watson–Crick/Sugar edge | ○–▷ | Parallel |
| 7 | *cis* | Hoogsteen/Hoogsteen | –■– | Anti-parallel |
| 8 | *trans* | Hoogsteen/Hoogsteen | –□– | Parallel |
| 9 | *cis* | Hoogsteen/Sugar edge | ■–▶ | Parallel |
| 10 | *trans* | Hoogsteen/Sugar edge | □–▷ | Anti-parallel |
| 11 | *cis* | Sugar edge/Sugar edge | –▶– | Anti-parallel |
| 12 | *trans* | Sugar edge/Sugar edge | –▷– | Parallel |

the use of • as a generic designation for non-Watson–Crick pairs in text. A set of black-and-white symbols to accurately specify each kind of non-Watson–Crick edge-to-edge pairing interaction was proposed, based on the use of three symbols to designate the interacting edges: *circles* for Watson–Crick edges, *squares* for Hoogsteen edges, and *triangles* for Sugar edges [12]. *Filled* and *open* symbols distinguish the *cis* and *trans* base-pairs. When the two interacting bases use the same edge, only one symbol is necessary (e.g. *cis* W.C./W.C or *trans* Hoogsteen/Hoogsteen). When an interaction involves two different edges, it is necessary to designate which edge corresponds to which base, e.g. 'A•G *cis* Watson–Crick/Hoogsteen' designates a pair in which the Watson–Crick edge of the A interacts with the Hoogsteen edge of the G. To distinguish the X•Y from Y•X pairs in such cases, a composite symbol is generated by linking the edge symbols by a line, as shown in Figure 2 and Table 1. Finally, a red or dotted arrow can be used to indicate points in the covalent chain at which reversals in strand orientation occur.

### Examples of 2D representations of RNA motifs

To illustrate these conventions, we present in Figure 3 examples of 2D representations of RNA motifs, starting with simple hairpin loops and proceeding to more complex motifs.

### Tetraloops

Figure 3(a) shows examples of recurrent hairpin motifs, taken from the structure of the 23S rRNA of *Haloarcula marismortui*, NDB file rr0033 [1]. The first two hairpins are essentially the same motif, although the base sequences differ. The diagram makes the similarity obvious. Both hairpin loops are closed by a 'sheared' (*trans* Hoogsteen/Sugar edge) base-pair, A808•G805 in one case and C256•U253 in the other. The *trans* Hoogsteen/Sugar edge pairs are designated with open symbols (indicating the *trans* geometry) consisting of squares, placed next to A808 or C256 (for the Hoogsteen edge), linked to triangles, placed next to G805 or U253 (for the Sugar edge). The strand polarity reverses direction immediately after

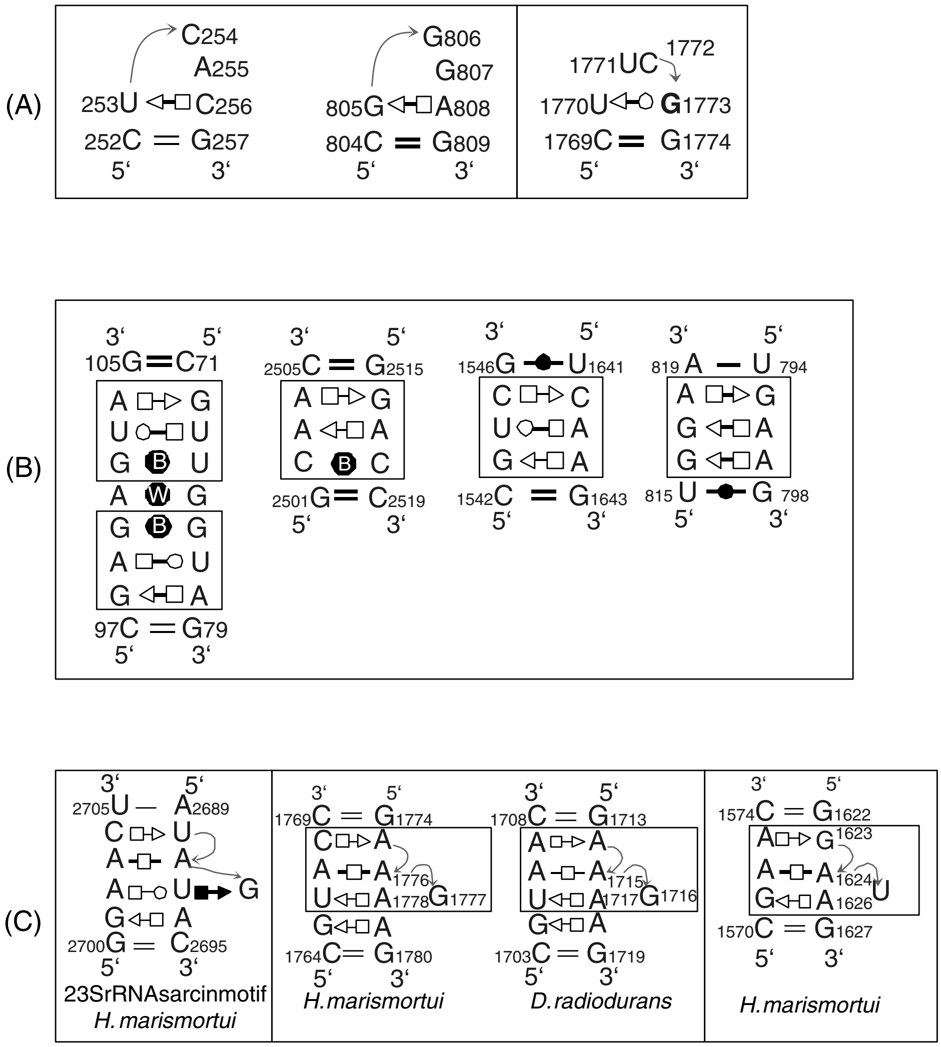

**Figure 3.** (A) Schematic representations of hairpin loops. (B) Bacterial loop E and related motifs. (C) Sarcin/ricin motif and related motifs

G805 or U253. Furthermore, the corresponding bases 806–808 in the first hairpin and 254–256 in the second are stacked as indicated by placing these bases one on the other. In fact, the two hairpins are superimposable in 3D space. By contrast, the third hairpin is very different and defines a different motif. The closing base-pair G1773•U1770 is *trans* Watson–Crick/Sugar edge and G1773 is in the *syn* configuration, indicated by the bold font. The strand reversal occurs between the third and fourth nucleotides of the hairpin loop (C1772–G1773). U1771 and C1772 are not stacked on each other.

### Symmetric internal loops

In Figure 3(b) we show examples of symmetric internal loops related to bacterial 5S Loop E submotifs. First, the complete bacterial loop E motif is shown. The boxes enclose the two submotifs and the annotations make clear that the sub-motifs are identical, since they each comprise a *trans* Watson–Crick/Hoogsteen pair flanked on one side by a *trans* Hoogsteen/Sugar edge base-pair and by a *cis* bifurcated pair on the other [6, 10]. A *cis* W.C. water-inserted G•A pair separates the two submotifs. A black disk with inscribed B indicates *cis* bifurcated pairs and a disk with

Copyright © 2002 John Wiley & Sons, Ltd.

inscribed W indicates the water-inserted *cis* W.C. A•G pair. Bifurcated pairs, in which a single exocyclic carbonyl or amino group of one base directly contacts the edge of a second base, and water-inserted pairs, in which single functional groups on each base interact directly, are intermediate between two of the standard geometries. Motifs related to the Loop E sub-motifs differ in the nature of the third base-pair, which is usually a *trans* Hoogsteen/Sugar edge pair, rather than a bifurcated pair. All are symmetric internal loops with cross-strand stacking of the conserved adenosines.

### Asymmetric internal loops

The next example (Figure 3c) is a motif related to the sarcin/ricin motif, a highly recurrent motif found throughout the ribosome world [11]. The sarcin/ricin motif also occurs in loop E of eukaryal 5S rRNA but should not be confused with bacterial loop E. An example of a sarcin/ricin motif is shown in Figure 3c (left), that of rat 28S rRNA, NDB file UR0002 [7]. The motif is an asymmetric 'internal loop' in which a local change in strand orientation occurs. The red arrows between U2690 and A2691 and between A2691 and G2692 indicate the local strand reversal that occurs at A2691. The positioning of A2691 above U2693 indicates the stacking between these two residues. The 'bulged' base, G2692, is actually hydrogen-bonded to U2693 and lies in the same plane as the U2693•A2702 *trans* W.C./Hoogsteen pair. This is indicated by placing all three bases on the same horizontal level on the page. The G2692•U2693 pair is *cis* Sugar-edge/Hoogsteen whereas the G2701•A2694 and U2690•C2704 pairs are *trans* Sugar-edge/Hoogsteen. We have identified a related motif in a highly conserved stem loop in Domain IV of 23S rRNA. The *H. marismortui* and *D. radiodurans* versions are shown in the middle panels of Figure 3c, which shows the similarities to the sarcin/ricin motif. The drawings helped us to identify a second independent occurrence of the motif in Domain III of 23S rRNA of *H. marismortui*. This is shown in the right-most panel of Figure 3c. A box is drawn around the conserved parts of the motif. Interestingly, the nucleotides corresponding to A1767 and C1768 participate in the RNA–RNA Bridge B5 and B6 identified in the 5.5 Å structure of the 70S ribosome, while the corresponding residues, A1572 and A1573, in the Domain II motif are involved in tertiary interactions as well.

### Conclusions

This geometrical classification of base-pairs is based on the observation that RNA bases can pair with each other using any of three distinct edges for hydrogen-bonding: the *Watson–Crick edge*, the *Hoogsteen edge* and the *Sugar edge* (which includes the 2′-OH and is also referred to as the *Shallow-groove edge*). Base-pairs can form with the glycosidic bonds of the nucleotides oriented either *cis* or *trans* relative to the base–base hydrogen bonds. Thus, 12 basic geometric families of base-pairs having at least two H-bonds connecting the bases are possible. For each geometric type, the relative orientations of the strands can easily be deduced. Several high-resolution examples in all 12 families are presently available [9]. Our annotation facilitates the recognition of isosteric relationships among base-pairs belonging to the same geometric family, and thus facilitates the recognition of recurrent 3D motifs from comparison of homologous sequences. Graphical conventions for accurately and unambiguously representing RNA motifs in secondary structure diagrams and in electronic databases have been defined.

This annotation facilitates the 2D representation of complex 3D structures, since conventions have been suggested for presenting the essential 3D features of RNA structures in a visually accessible and appealing 2D format. These include: (a) all canonical and non-Watson–Crick pairs; (b) changes in strand polarity in the folding of the RNA; (c) the occurrence of *syn* bases; and (d) essential stacking interactions. The nomenclature and classification were devised in order to facilitate the organization of the vast amount of new structural data so that, when properly stored, comparisons with homologous sequences and retrieval of motifs would be rapid and accurate.

### Acknowledgements

We thank Jesse Stombaugh for assistance with figure preparation (JS was supported by NSF-REU Grant CHE-9732463). This work was supported by NIH 2R15 GM55898 to NBL. EW wishes to thank the Institut Universitaire de France for support.

Copyright © 2002 John Wiley & Sons, Ltd.

### References

1. Ban N, Nissen P, Hansen J, Moore PB, Steitz TA. 2000. The complete atomic structure of the large ribosomal subunit at 2.4 A resolution [see comments]. *Science* **289**: 905–920.
2. Bartels H, Gluehmann M, Janell D, *et al.* 2000. Targeting exposed RNA regions in crystals of the small ribosomal subunits at medium resolution. *Cell Mol Biol (Noisy-le-grand)* **46**: 871–882.
3. Batey RT, Rambo RP, Doudna JA. 1999. Tertiary motifs in RNA structure and folding. *Angew Chem Int Ed Engl* **38**: 2326–2343.
4. Carter AP, Clemons WM, Brodersen DE, *et al.* 2000. Functional insights from the structure of the 30S ribosomal subunit and its interactions with antibiotics [see comments]. *Nature* **407**: 340–348.
5. Cech TR, Damberger SH, Gutell RR. 1994. Representation of the secondary and tertiary structure of group I introns. *Nature Struct Biol* **1**: 273–280.
6. Correll CC, Freeborn B, Moore PB, Steitz TA. 1997. Metals, motifs, and recognition in the crystal structure of a 5S rRNA domain. *Cell* **91**: 705–712.
7. Correll CC, Munishkin A, Chan YL, Ren Z, Wool IG, Steitz TA. 1998. Crystal structure of the ribosomal RNA domain essential for binding elongation factors. *Proc Natl Acad Sci USA* **95**: 13 436–13 441.
8. Harms J, Schluenzen F, Zarivach R, *et al.* 2001. High resolution structure of the large ribosomal subunit from a mesophilic eubacterium. *Cell* **107**: 679–688.
9. Leontis NB, Stombaugh J, Westhof E. 2002. The non-Watson–Crick base pairs and their associated isostericity matrices. *Nucleic Acids Res* **30**: 3497–3531.
10. Leontis NB, Westhof E. 1998a. The 5S rRNA loop E: chemical probing and phylogenetic data vs. crystal structure. *RNA* **4**: 1134–1153.
11. Leontis NB, Westhof E. 1998b. Conserved geometrical base-pairing patterns in RNA. *Q Rev Biophys* **31**: 399–455.
12. Leontis NB, Westhof E. 2001. Geometric nomenclature and classification of RNA base pairs. *RNA* **7**: 499–512.
13. Michel F, Jacquier A, Dujon B. 1982. Comparison of fungal mitochondrial introns reveals extensive homologies in RNA secondary structure. *Biochimie* **64**: 867–881.
14. Westhof E. 1992. Westhof's rule [letter]. *Nature* **358**: 459–460.
15. Westhof E, Fritsch V. 2000. RNA folding: beyond Watson–Crick pairs. *Structure Fold Des* **8**: R55–65.

Copyright © 2002 John Wiley & Sons, Ltd. 
